# Tracking the Initial Diffusion of SARS-CoV-2 Omicron Variant in Italy by RT-PCR and Comparison with Alpha and Delta Variants Spreading

**DOI:** 10.3390/diagnostics12020467

**Published:** 2022-02-11

**Authors:** Valerio Caputo, Giulia Calvino, Claudia Strafella, Andrea Termine, Carlo Fabrizio, Giulia Trastulli, Arcangela Ingrascì, Cristina Peconi, Silvia Bardini, Angelo Rossini, Antonino Salvia, Giovanna Borsellino, Luca Battistini, Carlo Caltagirone, Raffaella Cascella, Emiliano Giardina

**Affiliations:** 1 Genomic Medicine Laboratory UILDM, IRCCS Santa Lucia Foundation, 00179 Rome, Italy; v.caputo91@gmail.com (V.C.); giulia.calvino95@gmail.com (G.C.); claudia.strafella@gmail.com (C.S.); giulia.trastulli95@gmail.com (G.T.); arcangelaingrasci@gmail.com (A.I.); cristinapeconi@gmail.com (C.P.); bardinisilvia99@gmail.com (S.B.); raffaellacascella@virgilio.it (R.C.); 2 Department of Biomedicine and Prevention, Tor Vergata University, 00133 Rome, Italy; 3 Data Science Unit, IRCCS Santa Lucia Foundation, 00179 Rome, Italy; andreatermine544@gmail.com (A.T.); carlo.fabrizio217@gmail.com (C.F.); 4 Medical Services, IRCCS Santa Lucia Foundation, 00179 Rome, Italy; a.rossini@hsantalucia.it (A.R.); a.salvia@hsantalucia.it (A.S.); 5 Neuroimmunology Unit, IRCCS Santa Lucia Foundation, 00179 Rome, Italy; g.borsellino@hsantalucia.it (G.B.); l.battistini@hsantalucia.it (L.B.); 6 Department of Clinical and Behavioral Neurology, IRCCS Santa Lucia Foundation, 00179 Rome, Italy; c.caltagirone@hsantalucia.it

**Keywords:** omicron variant, COVID-19, molecular diagnosis, naso-oropharyngeal swabs, RT-PCR, SARS-CoV-2, rapid screening, VOC, spreading

## Abstract

The emergence of the Omicron SARS-CoV-2 variant caused public health concerns worldwide, raising the need for the improvement of rapid monitoring strategies. The present manuscript aimed at providing evidence of the utility of a diagnostic kit for the routine testing of SARS-CoV-2 infection as a cost-effective method for tracking the Omicron variant in Italy. The study was conducted on patients’ naso-oropharyngeal-swab-derived RNA samples. These samples were subjected to RT-PCR using the TaqPath COVID-19 RT PCR CE IVD kit. Nonparametric testing and polynomial models fitting were used to compare the spreading of Alpha, Delta and Omicron variants. The samples of interest were correctly amplified and displayed the presence of S gene-target failure, suggesting that these patients carry the Omicron variant. The trend of diffusion was found to be significantly different and more rapid compared with that of the Alpha and Delta variants in our cohorts. Overall, these results highlight that the S gene target failure was a very useful tool for the immediate and inexpensive tracking of Omicron variant in the three weeks from the first detection. Thus, our approach could be used as a first-line screening to reduce the time and costs of monitoring strategies, facilitating the management of preventive and counteracting measures against COVID-19.

## 1. Introduction

The emergence of novel and more infectious SARS-CoV-2 variants of concerns (VOCs) raises several issues because of their rapid spreading, increased transmission risk and their potential escape to immune system responses that could reduce the action of vaccines [1]. Recently, the omicron variant (oV, lineage B.1.1.529) has come to worldwide attention given its increased transmissibility and expected rapid diffusion [2]. Therefore, the rapid tracking of VOCs, and especially of oV, during the diagnostic routine could be very useful to estimate the real trend of variant diffusion.

To date, the real-time PCR (RT-PCR) technology represents the gold standard for the accurate SARS-CoV-2 infection and comprehensively showed high reliability for the viral diagnosis given the higher sensitivity and specificity compared with other analytical tools, such as antigen testing [3,4,5]. Moreover, this technology coupled with the TaqPath COVID-19 RT PCR CE IVD kit (Thermo Fisher Scientific, Waltham, MA, USA) has also allowed to discriminate the Alpha variant (αV, lineage B.1.1.7) during its spreading from December 2020. In fact, this variant harbored a six nucleotides deletion within S gene (HV69-70del) that has been found responsible for the S Gene Target Failure (SGTF) using this kit [6].

Intriguingly, sequencing data on oV carriers highlighted the occurrence of the HV69-70del [2], as well, suggesting that the utilization of this kit could enable the detection of oV. Herein we provide hands-on evidence of the ability of TaqPath COVID-19 RT PCR CE IVD kit (Thermo Fisher Scientific) to identify infected samples that are likely to carry oV and of its utility as a cost-effective rapid method for oV tracking. On this subject, we were able to detect 522 oV-suggestive samples during the routinary molecular diagnosis provided by the COVID-19 Laboratory at the Scientific Institute for Research, Hospitalization and Healthcare (IRCCS, San Giovanni Rotondo, Italy) Santa Lucia Foundation from 13 to 31 December 2021 in Rome, Lazio, Italy. Moreover, we were able to analyze the initial trend of diffusion of suspect oV among the other positive samples, comparing it with the spreading of the previous predominant VOCs in Italy, namely the αV and Delta variant (δV, lineage B.1.617.2) in our recent and past cohorts, respectively.

## 2. Materials and Methods

### 2.1. Molecular Diagnosis of SARS-CoV-2

The patients were admitted to the COVID-19 Laboratory of Santa Lucia Foundation IRCCS on the basis of clinical suspects and/or known contact to positive subjects. The research was approved by the local Ethics Committee and was performed according to the Declaration of Helsinki. Naso-oropharyngeal samples were obtained using cotton swabs in Universal Transport Medium (UTM) (Copan Diagnostics, Murrieta, CA, USA). The presence of SARS-CoV-2 was assessed by extracting viral RNA from 300 μL of UTM through Magpure virus DNA/RNA purification kit (Hangzhou Bigfish Bio-tech Co., Ltd., Hangzhou, China) on the automated extraction systems Nuetraction 32 and 96 Nucleic Acid Purification Systems (Hangzhou Bigfish Bio-tech Co., Ltd.). The extracted RNAs were then subjected to one-step real-time PCR (RT-PCR) by TaqPath COVID-19 RT PCR CE IVD kit (Thermo Fisher Scientific, Waltham, MA, USA) on QuantStudio 5 RT-PCR system according to manufacturer’s instructions. In particular, this kit includes specific Taqman probes for the detection of *S*, *N* and *Orf1ab* genes. The resulting data were analyzed and interpreted using the QuantStudio DA2 and COVID-19 Interpretive Softwares (Thermo Fisher Scientific).

### 2.2. Statistical Analyses

In order to evaluate the diffusion of αV, δV and oV, the ratio of variant carriers over the total number of positive patients detected was obtained for each day. A heteroscedastic one-way ANOVA for trimmed means with trim level = 0.1 and α = 0.05 was used to assess differences in the diffusion between oV and αV, as well as oV and δV across 18 days since their first detection. The obtained *p*-value (*p*) was corrected by the false discovery rate (FDR) and the effect size was computed along with the Confidence Intervals (CI) by bootstrapping with *n* = 100 (days) and the bootstrapped effect size was interpreted using Cohen’s table [7]. In order to assess the best fitting functions to the distribution of αV, δV and oV data, linear and 2nd grade polynomial regressions were used to predict the percentage of variant positives. Subsequently, the best fit was evaluated by computing R^2^, and function equations were extracted. All of the statistical analyses were performed using the R v.4.0.1 language and WRS2 library [8,9,10].

## 3. Results and Discussion

**Figure 1:**
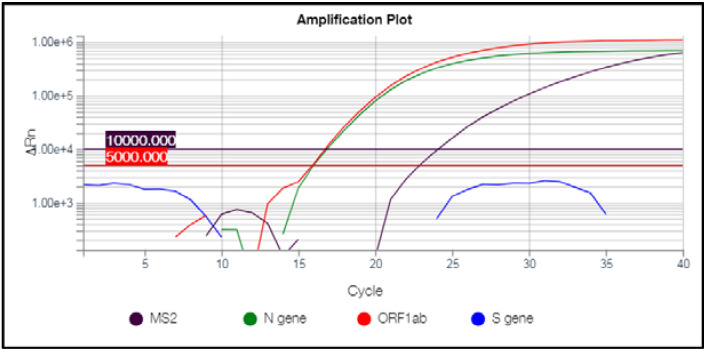
Amplification plot of a suspect omicron variant carrier.The amplification plot related to a sample suggestive of being oV is shown, as visualized on QuantStudio DA2 Software. In particular, only *N* and *Orf1ab* genes were successfully amplified, whereas the amplification of the *S* gene failed. The resulting Ct values are calculated based on the threshold of 5000.00 ∆ normalized reporter (Rn) value according to manufacturer’s instructions. The MS2 represents the probe for the detection of the internal control provided by the kit and its related Ct is obtained based on the threshold of 10,000.00 ∆Rn, as indicated by the manufacturer.

The 522 samples of interest were characterized by a peculiar pattern of amplification, namely the presence of only *N* and *Orf1ab* targets, which were detected at low Ct values ranging from 10 to 21 and from 11 to 23, respectively, and SGTF (Figure 1).

These results were compatible with a high viral load (>7 log10 copies/mL), often indicative of an early phase of infection. This same pattern of amplification occurred in presence of αV and allowed us to distinguish the αV from the pre-existing strains during the first months of 2021 [11]. Considering that the δV lacks this deletion and that the detection of αV is nowadays negligible, the above-mentioned samples characterized by the SGTF were suggestive of being infected by oV. Overall, we found that the presence of viral genome was correctly detected without loss of performance, as shown by the correct amplification of *N* and *Orf1ab* genes. Therefore, the detectability of virus was not impaired, ensuring a correct diagnosis of positivity to SARS-CoV-2 for these infected patients. Moreover, the occurrence of SGTF allowed for the distinguishing these samples from other positive samples with the amplification pattern typical of δV (showing the amplification of all three targets). Our laboratory sent 41 samples displaying SGTF for molecular surveillance to the INMI Lazzaro Spallanzani (Rome, Italy) in accordance with the established indications. All these samples were confirmed to be carriers of oV.

**Figure 2:**
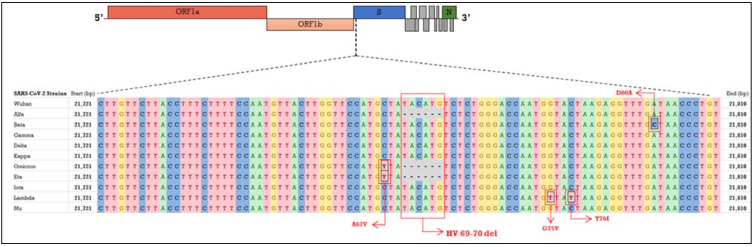
Focused alignment of the genomic sequence encompassing the HV69-70del within the *S* gene. The SARS-CoV-2 genome has been schematized and the genes/regions targeted by the TaqPath COVID-19 RT PCR CE IVD kit, namely *Orf1ab*, *S* and *N* have been highlighted. The nucleotide sequence encompassing the HV69-70 deletion and 40 bp upstream and downstream within the S gene is shown and aligned among the VOCs (Alpha, Beta, Gamma, Delta and Omicron) and other variants of interest (Eta, Iota, Kappa, Lambda and Mu). The occurrence of the HV69-70del responsible for the SGTF can be visualized in Alpha and Omicron among the VOCs and in Eta, among the related variants. Other mutations characterizing viral variants which occurred within the focused sequence are shown as well. The Wuhan original strain was used as reference genome and the related sequence was retrieved from NCBI (NC_045512.2, https://www.ncbi.nlm.nih.gov, accessed on 8 February 2022). Data on variants’ mutational landscape were obtained from Nextrain.org, CoVariants (https://covariants.org/variants/, accessed on 8 February 2022) and UCSC (https://genome.ucsc.edu, accessed on 8 February 2022).

Indeed, we were able to observe a rapidly increasing trend of suspect oV carriers over the total positive samples (from 13 December to 17 December, these cases represented the 1.5% of total positive samples and reached an average of 39% during the last week of December). We compared this trend of diffusion with that related to the αV carriers detected from December 2020 and to the δV carriers detected from June 2021. Importantly, δV displayed the same amplification pattern of other VOCs, such as Beta (B.1.351) or Gamma (P.1) variants, and in general, of viral strains that do not harbor the HV69-70del, which does not allow their discrimination by means of our approach (Figure 2).

**Figure 3:**
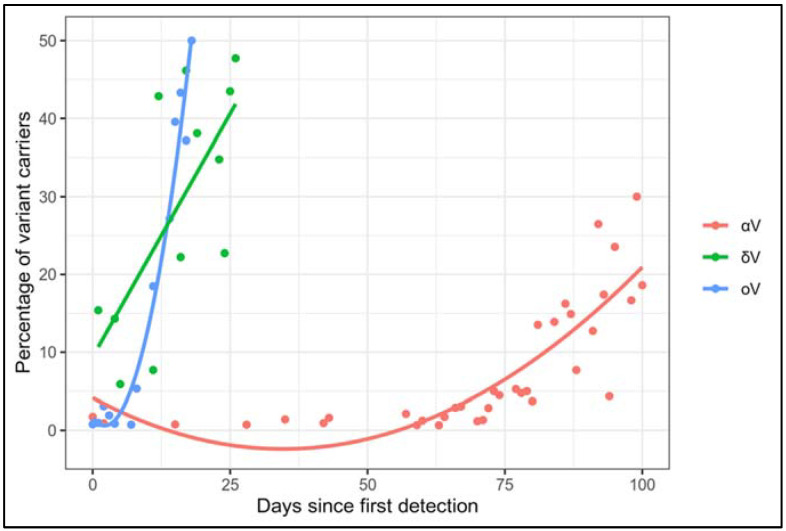
Comparison between the diffusion of oV versus αV and δV in our cohort’s patients. The diffusion of αV and oV in our cohorts is described by different functions (αV: *y* = 0.00005355*x*^2^ − 0.0085694*x* + 0.0153644, oV: *y* = 0.0019871*x*^2^ − 0.003777*x* + 0.04147; δV: y=0.01248x+0.09412). The solid lines represent the 2nd degree polynomial regressions for αV and oV and the linear regression for δV.

We found significant differences in their rates (oV vs. αV *p* = 2.962 × 10^−2^, oV vs. δV *p* = 2.811 × 10^−2^) with a moderate effect size (oV vs. αV effect size = 0.5, CI = 0.42–2.10; oV vs. δV effect size = 0.44, CI = 0.30–0.80) in our cohorts. Moreover, αV and oV distributions were modeled by different 2nd grade polynomial regression functions characterized by adjusted R^2^ of 0.96 and 0.70 for oV and αV, respectively, whereas δV was modeled by a linear function, with an adjusted R^2^ of 0.44. These functions displayed strongly different steeps (namely, 2nd grade terms coefficients equal to 1.9871 × 10^−3^ and 5.455 × 10^−5^ for oV and αV, respectively, and the coefficient related to δV was equal to 1.248 × 10^−2^) (Figure 3) which further support the different trends of diffusion. The different modeling of δV could be due to the available lower sample size with respect to oV and αV. As a matter of fact, during the initial period of δV diffusion, from June 2021 to the start of July 2021 our laboratory performed a lower number of diagnostic tests, which mirrored the national trend in the same period [12].

Overall, based on the statistical analysis related to the comparisons, the oV displayed a more rapid spread in our cohorts (Figure 3), in line with reported evidence that found a more rapid diffusion of oV than previous VOCs in other populations [13,14]. Indeed, the higher rate of diffusion of oV could be due to the novel variant’s features but also to social and individual behavior, since the “lockdown” established during the first months of 2021 and progressively attenuated from the summer of 2021 could have slowed the spreading of αV. Overall, our observation raised the emerging need for an accurate and timely evaluation of the real prevalence of oV and its speed of diffusion in the country.

Altogether, this evidence highlights that the multi-target approach related to the employed kit was very useful for the timely identification of possible oV carriers without the need of additional analyses respect to those already used for the diagnostic routine.

Our approach was therefore used as a first-line screening to track the circulation of this emerging variant (which was reported to has a 0.19% prevalence in Italy at 16 December and to have reached the 21% on 20 December [15]) and improve the selection of samples to be sequenced for monitoring and surveillance programs in Italy. In fact, the high cost of the Whole Genome Sequencing (WGS) of a large number of samples and the related difficulties in their processing may impact the public health system, leading to significant delays in providing crucial information for arranging eventual preventing measures [16]. Given this, RT-PCR is starting to be proposed as a helpful method for the rapid identification of circulating variants, using specific assay targeted to genetic alterations known to characterize viral strains [17]. A preliminary screening performed within the diagnostic routine would be even more convenient to detect possible oV carriers, since it would be performed without additional assays, time and costs apart from those related to the routinary analyses.

By this method, a rapid and effective strategy for monitoring the circulation of oV could be pursued at the same time as the molecular diagnosis. Such timely assessment will also be fundamental after the initial diffusion, in order to evaluate the real impact on hospitalization and on the effectiveness of vaccines, facilitating the management of the immunization campaign and providing adequate counteracting measures against COVID-19.

## Data Availability

The data described in the present study are included within the manuscript.
